# The Interplay between *Salmonella* and Intestinal Innate Immune Cells in Chickens

**DOI:** 10.3390/pathogens10111512

**Published:** 2021-11-19

**Authors:** Adil Ijaz, Edwin J. A. Veldhuizen, Femke Broere, Victor P. M. G. Rutten, Christine A. Jansen

**Affiliations:** 1 Division of Infectious Diseases and Immunology, Department of Biomolecular Health Sciences, Faculty of Veterinary Medicine, Utrecht University, Yalelaan 1, 3584 CL Utrecht, The Netherlands; a.ijaz@uu.nl (A.I.); e.j.a.veldhuizen@uu.nl (E.J.A.V.); f.broere@uu.nl (F.B.); v.p.m.g.rutten@uu.nl (V.P.M.G.R.); 2 Department of Veterinary Tropical Diseases, Faculty of Veterinary Science, University of Pretoria, Onderstepoort, Pretoria 0110, South Africa; 3 Cell Biology and Immunology Group, Department of Animal Sciences, Wageningen University & Research, De Elst 1, 6708 PB Wageningen, The Netherlands

**Keywords:** chicken, *Salmonella*, GALT, innate immune cells, intestine

## Abstract

Salmonellosis is a common infection in poultry, which results in huge economic losses in the poultry industry. At the same time, *Salmonella* infections are a threat to public health, since contaminated poultry products can lead to zoonotic infections. Antibiotics as feed additives have proven to be an effective prophylactic option to control *Salmonella* infections, but due to resistance issues in humans and animals, the use of antimicrobials in food animals has been banned in Europe. Hence, there is an urgent need to look for alternative strategies that can protect poultry against *Salmonella* infections. One such alternative could be to strengthen the innate immune system in young chickens in order to prevent early life infections. This can be achieved by administration of immune modulating molecules that target innate immune cells, for example via feed, or by in-ovo applications. We aimed to review the innate immune system in the chicken intestine; the main site of *Salmonella* entrance, and its responsiveness to *Salmonella* infection. Identifying the most important players in the innate immune response in the intestine is a first step in designing targeted approaches for immune modulation.

## 1. Introduction

Salmonellosis is an intestinal bacterial infection of poultry. The disease may be caused by various serovars of *Salmonella* and infection can lead to severe symptoms such as gastroenteritis, septicemia, and typhoid fever and can cause mortality in young chickens. This makes *Salmonella* infection a major concern for the poultry industry [1]. The severity of infection depends on the serovar, the history of exposure, the age and the genotype of chickens. Some of the *S. enterica* serovars, such as *S.* ser. Gallinarium and *S.* ser. Pullorum, are host specific, but the majority of the *Salmonella* strains can infect multiple host species. Colonization of *S.* ser. Enteriditis and *S.* ser. Typhimurium in the ileum and cecum of the chicken causes enteric salmonellosis in young chickens of 2–3 days of age. *S*. ser. Pullorum and *S*. ser. Gallinarum infect chickens of all ages and cause a chronic typhoid-like disease resulting in significant mortality. *S. enterica* serovars induce systemic infection via lymphoid tissues such as Peyer’s patches and cecal tonsils. *Salmonella* infected phagocytes enter the lymphatics and bloodstream and disseminate bacteria to spleen, liver, bone marrow, and ovaries, thereby causing a second round of infection. In chickens, *S. enterica* serovars may reside in the spleen for months without showing obvious clinical signs and lead to infection of the reproductive tract [2]. Especially, *S*. ser. Pullorum colonizes the reproductive tract of the chickens and infects progeny through vertical transmission [3]. These examples clearly show the large diversity of infections and related disease caused by different *Salmonella* serovars, and thereby the complexity to describe and define a ‘*Salmonella* infection’.

*Salmonella* contaminated poultry meat products and eggs from *Salmonella*-infected chickens can lead to food-borne zoonosis in humans as poultry products are highly consumed food commodities of humans [4,5,6]. In 2010, more than 0.5 billion *S*. ser. Enteriditis contaminated eggs were recalled because of a nationwide outbreak of human food-borne salmonellosis in the US (https://www.cdc.gov/Salmonella/2010/shell-eggs-12-2-10.html, accessed on 26 August 2021). A recent report mentioned 87,923 confirmed cases of human salmonellosis in 2019, the 2nd highest human zoonosis in Europe [6]. In the EU, the overall economic burden due to human salmonellosis has been estimated to be over three billion euros annually (https://www.efsa.europa.eu/en/topics/topic/Salmonella, accessed on 26 August 2021). The worldwide incidence of salmonellosis is 1.3 billion, due to which 3 million people die annually [7]. Hence, controlling *Salmonella* infections in chickens is highly important to diminish the incidence of zoonotic *Salmonella* infections in humans.

To control *Salmonella* infections, the EU has adopted an integrated approach mainly focused on food safety by ensuring pathogen elimination from animal food products, including poultry meat and eggs (https://eur-lex.europa.eu/eli/reg/2012/1190/oj, accessed on 26 August 2021). The EU member states have initiated a *Salmonella* control program in poultry aiming to implement strict biosecurity, using vaccination against *Salmonella* in poultry, and destruction of infected eggs and birds in case of outbreaks in flocks (https://eur-lex.europa.eu/eli/reg/2006/1177/oj, accessed on 30 August 2021).

In poultry production, vaccination against *Salmonella* is nowadays common practice to provide protection to young birds. However, multiple studies have shown that vaccination doesn’t guarantee full protection and prevention of bacterial shedding in older birds, especially in layers. Moreover, *Salmonella* vaccines are not multivalent, so whereas those vaccines help to limit the spread of certain *Salmonella* serovars, they allow other harmful *Salmonella* strains to grow. Therefore, even after vaccination, a flock can be infected by *Salmonella* and transmit pathogen both horizontally and vertically.

Antibiotics as feed additives have proven to be an effective prophylactic option to control intestinal *Salmonella* infections [8], but due to limited effectiveness of antibiotics against *Salmonellae* and resistance issues in humans and animals, the use of antimicrobials has been banned in food animals in Europe [9]. Hence, there is an urgent need for alternative strategies to protect poultry from *Salmonella* infection. One alternative is to strengthen the innate immune system in young chickens to prevent early life intestinal infections. The focus on young chickens is specifically important because their adaptive immune system is not fully developed to generate an effective *Salmonella* specific immune response and therefore host defense mostly relies on innate immunity. Strengthening of the innate immune system in young chickens can, for example, be achieved by addition of immune modulating molecules to feed, or even by in-ovo application of immunostimulants.

Innate immunity that plays an important role against *Salmonella* invasion, may be affected by several factors. In chickens, the differentiation and proliferation of innate immune cells start during embryonic life and continue during the early days post-hatch, in addition, it has been shown that the competence of innate immune cells improves with increasing age [10]. Furthermore, the intestinal microbiota do contribute to innate immunity as it provides competition to the invading *Salmonella* serovars in the gut. Moreover, feed helps to attain diversity of intestinal microbiota that contribute to resistance to *Salmonella* colonization in the gut [11]. Also, the genetic background of the birds, as well as housing environment can affect innate immunity in chickens [12,13].

The objective of this review is to discuss the chicken intestinal innate immune system in order to determine how innate immune fitness can be increased and used to prevent infectious diseases like salmonellosis in chickens and hence to reduce the risk of zoonotic infections in humans.

## 2. The Chicken Intestine

**Figure 1:**
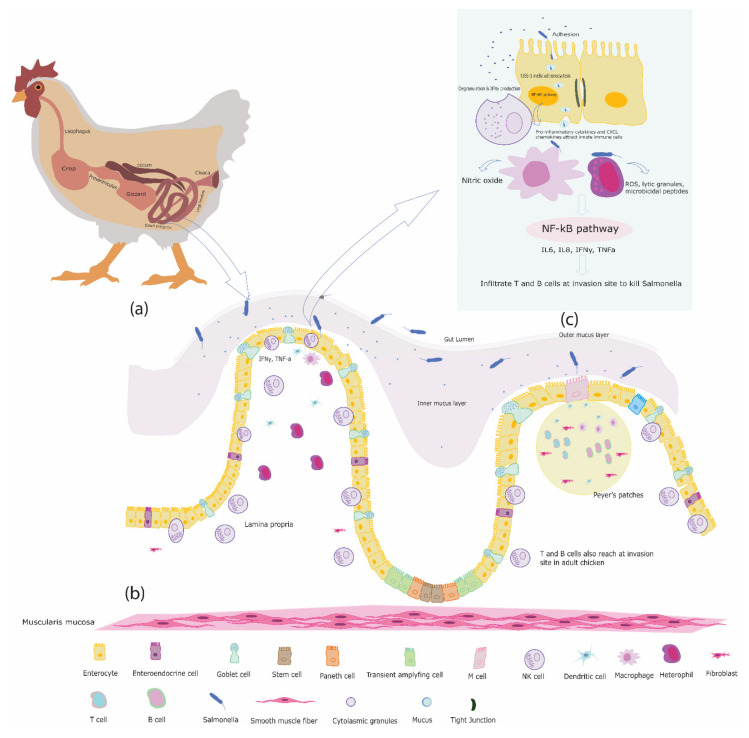
(**a**) Schematic representation of the chicken gastrointestinal tract, including the avian specific crop, gizzard, ceca, and cloaca; (**b**) Anatomical features of chicken intestinal epithelium and innate immune cells embedded in intestinal epithelium, lamina propria, and Peyer’s patches; (**c**) Cross-talk between host innate immune cells and *Salmonella* serovars.

The intestine is a complex organ that primarily functions to digest food into small particles and to transport it into the body to produce energy [14]. The chicken gut is home of a diverse microbial community, most of which are Gram-positive and facultative anaerobes [15]. The chicken gastrointestinal (GI) system is comparable to the digestive tract of mammals, but with several avian specific features like the crop, gizzard, ceca, and cloaca [14] (Figure 1a). The avian GI tract starts from the beak connected to the stomach by the esophagus. The crop, an out-pocket of the esophagus, used to store and process food for digestion [16], does so, due to its own microbiota, which includes *Furmicutes*, *Bacteriodetes*, *Proteobacteria*, and *Actinobacteria* [17]. The chicken stomach consists of the proventriculus, which is a glandular part, and the ventriculus or gizzard, which acts as a mechanical stomach because of its strong muscles and grinding action. The gizzard also acts as a microbial barrier because of its low pH [18]. The gizzard is followed by the small intestine, which consists of three sections, the duodenum, jejunum, and ileum, respectively. The duodenum plays an important role in food digestion. Bicarbonate and digestive enzymes from the pancreatic ducts, and bile from the liver help in food digestion. The absorption of nutrients takes place in the ileum. The most predominant bacterial species present in all three sections of the small intestine is *Lactobacillus* [18,19]. The Meckel’s diverticulum is a small structure present on the small intestine at the transition between jejunum and ileum. The ceca are two blind pouches attached to the small intestine, and involved in the fermentation of nutrients [20]. The cecal microbiota has greater richness and diversity as compared to the small intestinal microbiota [18,21,22]. It consists of *Enterococcus*, coliforms, *Lactobacillus*, and yeast [23,24]. The large intestine in chickens, the colon, terminates at the cloaca, which is a common cavity for the digestive and urogenital tract [14].

In the early days post-hatch, the intestine of the chickens is relatively sterile since the gut microbiota is still developing, which will result in 10^10^ bacteria/gram of digesta in the ileum and cecum, within 3 days [25]. During the early days, the chicken gut provides relatively low competitive exclusion for pathogen colonization and growth. Thus, it is a favorable site for *Salmonella* colonization [26,27]. External factors such as food and water can be a source of transferring *Salmonella* [28,29]. At this age exposure to *Salmonella* is detrimental to the growth of the natural gut microbiota, by causing reduction in microbial diversity, and hence allowing the growth of opportunistic pathogens in the gut [30]. The gut and its associated lymphoid tissue (GALT) provide protection against harmful effects of intestinal pathogens like *Salmonella.*

## 3. The Immune System of the Chicken Intestine

The chicken intestinal epithelium is a monolayer consisting of various cell types. Intercellular junctional complexes, tight junctions, present between the intestinal epithelial cells maintain the gut barrier integrity. Tight junctions hold the intestinal epithelial cells together to prevent the entry of enteric pathogens, for example, *Salmonella*, to the lamina propria, and thus maintain gut homeostasis [31]. In addition to the gut microbiota, which inhibits pathogen colonization; intestinal epithelial cells through their secretions also inhibit pathogen entry in the gut. For example, the outer surface of the intestinal epithelial cells is covered with a mucus layer, which provides a diffusive barrier between the intestinal microbiota and epithelial cells [32]. Mucus is secreted by intestinal goblet cells and is composed of 95% water and only 5% of the mucus contents contain salts, lipids, gel-forming mucin glycoproteins, defensins, and lysozyme [33,34,35,36,37,38]. Muc2, Muc5a, Muc5b, and Muc6 are gel forming mucins in chicken intestine [39]. The intestinal epithelium is the interface between the gut microbiota and the body [40]. The most abundant cell type of the intestinal epithelium, the enterocytes, represent more than 80% of the intestinal lining [41]. The primary function of the enterocytes is to digest and absorb food. Besides enterocytes, the monolayer contains the goblet cells, which secrete mucus and are abundantly present in the ileum (26%) and jejunum (23%) [42]. A third cell type present is the enteroendocrine cells, whose main function is to secrete enzymes to promote digestion of food components. These cells also produce hormones that are released in the bloodstream instead of the intestinal lumen. Enteroendocrine cells are sparsely present in the intestinal epithelium representing approximately 1% of the cells [43]. Paneth cells, specialized intestinal epithelial cells located at the base of the intestinal crypts of most of the vertebrates, are best known for their secretion of antimicrobial compounds such as phospholipase, lysozyme and α-defensins [44]. Three types of Paneth cells have been identified so far in poultry. These include, c-type -conventional or chicken type- mostly present in small intestine, and oviduct [45], g-type -goose type-, which are prevalent in the lungs and bone marrow [46], and g2-type which are expressed in the liver, kidney, and small intestine [45]. In chickens, however, the presence of the Paneth cells is controversial [45].

In addition to the gut epithelial cells, also immune cells are present along the length of the intestine as the GALT, part of the mucosa-associated lymphoid tissues (MALT) [47]. In contrast to mammals, chickens do not have encapsulated lymph nodes, instead mucosa-associated and diffused lymphoid tissues are present. The GALT is exposed to more antigens than any other tissue of the body and is present at strategically important locations along the length of the intestine. The chicken GALT includes Peyer’s patches (PP), cecal tonsils (CT), Meckel’s diverticulum, intraepithelial lymphocytes (IELs), and lamina propria lymphocytes (LPL), which are located beneath the intestinal epithelium as shown in Figure 1b [48,49,50].

The cellular architecture of the chicken GALT primarily consists of IELs (γδ T cells, αβ T cells, and NK cells), and phagocytic cells such as dendritic cells (DCs), and monocytes/macrophages. These cells either directly kill the invading pathogens or secret pro-inflammatory cytokines to attract other immune cells to mount the host immune response. DCs and macrophages also act as antigen presenting cells in the secondary lymphoid organs such as PP [51]. The immune cells of the GALT work in collaboration to initiate host defense responses during bacterial invasion. The innate immune cells are armed with sensors, specific for pathogens, so-called pattern recognition receptors (PRRs) [52]. The PRRs recognize pathogen-associated molecular patterns (PAMPs) and damage-associated molecular pattern (DAMPs). The PRRs can be cell-associated (Toll-like receptors, scavenger receptors, C-type lectins, intracellular receptors) or soluble components (pentraxins, LPS binding proteins, collectins). The PRRs are present at the cell surface in the plasma membrane, and in the cytoplasm and recognize most of the enteric pathogens. Probably the most important, or at least the best described PRRs are Toll-Like Receptors (TLR). These sensory receptors recognize pathogens through ligands present on bacteria (and/or other pathogens), which include, for example, flagellin (TLR5), LPS (TLR4), and CpG-DNA (TLR21) [53,54,55,56,57].

## 4. Innate Immune Cells in the Chicken Intestine

In the intestine, the IELs are embedded between the enterocytes of the intestinal epithelium. In adult chickens, the IEL population is comprised of NK cells, γδ, and αβ T cells [58,59]. Among the IEL population, NK and γδ T cells are innate immune cells and formulate the first line of host immune defense against invading pathogens [60]. An overview of the different mediators produced by intestinal innate immune cells as well as the intestinal epithelial cells, and the functions performed by them is shown in Table 1.

### 4.1. Natural Killer (NK) Cells of the Intestinal Immune System

NK cells are involved in the innate defense, they are scavenger cells and their primary function is to recognize the virally infected, transformed, and neoplastic host cells and kill them [61,62]. NK cells originate in the bone marrow and migrate towards blood and organs like the spleen, lungs, and intestine [63]. In the chicken intestine NK cells are embedded between the enterocytes as intraepithelial lymphocytes along the length of the gut. In intestinal intraepithelial lymphocytes NK cells comprise even higher numbers than CD8^+^ T cells and γδ T cells [59,64] suggesting an important role in the intestinal innate immune system [65].

Chicken NK cells are described as a population of cells that lack surface expression of CD3 or Ig [64] that is able to kill the NK susceptible cell line (LSCC-RP9) [59]. Based on these characteristics, many studies have reported the presence of NK cells in various tissues like spleen, lungs and intestine of chickens [66,67,68,69,70]. NK cells are also present in large numbers in the embryonic spleen and duodenum of chickens [59,67,70,71] indicating that they are indeed important in early life innate defense.

The induction of NK cell activation is mediated by an array of activating and inhibitory receptors which are present on their surface [72]. Also, in the chicken genome NK cell receptors have been reported to be present. The leucocyte receptor complex on chromosome 31 encodes for chicken Ig-like receptors (CHIR) [73]. Chromosome 1 harbors the NK gene complex which encodes NKG2/CD94 and syntenic to the mammalian NK complex region [73,74,75].

Upon crosslinking, activation receptors on NK cells signal via immunoreceptor tyrosine-based activation motifs (ITAMs) which initiates downstream signaling, while inhibitory receptors signal via immunoreceptor tyrosine-based inhibitory motifs (ITIMs). The delicate balance between activating and inhibitory signals received by NK cell receptors activates NK cells [76,77,78].

Once activated, NK cells release lytic granules that contain perforin and granzyme to lyse target cells [79,80]. The degranulation of NK cells upon activation can be detected using the CD107 assay [67], since enhanced surface expression of CD107a, the lysosomal-associated membrane protein-1 (LAMP-1), is associated with NK cell activation [66]. In chickens, enhanced CD107 surface expression was reported on CD3 negative cells that express surface markers such as 28-4, 20E5, CD11b/c, and 7C1 suggesting that these are markers of cells with NK cell function.

NK cells that express 28-4 (the chicken IL-2Ra orthologue) are mostly present in the duodenum of layer chickens and have been shown to able to kill susceptible target cells (LSCC-RP9) [59,81]. In other organs such as spleen, blood, and lung of layer chickens NK cells expressing 28-4 are less dominant, here the major populations express 5C7 and 20E5 [59,67,81,82].

In the intestine, the majority of NK cells express 28-4, while lower expression of NK cell markers 20E5 and 5C7 was observed. The number of 28-4 expressing NK cells was higher at day 1 post-hatch compared to levels at embryonic day 14 and 18, and remained stable throughout the life. Conversely, the number of 20E5^+^ and 5C7^+^ cells was lower in 1 day-old chickens compared to embryonic day 14 and 18 and increased until day 21 post-hatch. Among intestinal NK cells, surface expression of CD107 was also higher in the 28-4^+^ subset as compared to intestinal 20E5^+^ cells suggesting that this population may play a role in the intestinal innate immune response [70].

Upon activation NK cells may also secrete the cytokine interferon-γ, which recruits and activates immune cells from peripheral blood and nearby lymphoid tissues to amplify the innate immune response against bacteria and/or viruses and to initiate adaptive immune response [83,84].

### 4.2. Heterophils of the Intestinal Immune System

Heterophils are the avian analogues of mammalian neutrophils and immediately appear at the site of infection to eliminate invasive pathogens [85,86]. Heterophils use multiple strategies such as phagocytosis, degranulation, and oxidative burst to kill the pathogens. In chickens, heterophils are the predominant granulocytes in circulating blood and the gut [87,88,89,90,91,92]. Heterophils are comparatively higher in number in young chickens as compared to the older ones [93]. However, in young birds heterophils are functionally less active as indicated by decreased phagocytosis, degranulation, oxidative burst and, subsequently, killing of bacteria.

In the chicken intestine, pro-inflammatory cytokines produced as a result of microorganism invasion attract heterophils to the site of infection [94]. Upon contact with pathogens, heterophils are activated through the interaction of TLRs with bacterial ligands such as LPS, peptidoglycan, flagellin, and lipoteichoic acid [53,95]. This activation of heterophils results in a sequence of events including phagocytosis, oxidative burst, degranulation, and cytokine (IL1β, IL6) and chemokine (CXCLi2) production through the NF-κB pathway [53,56]. Heterophils also become activated via avian triggering receptor expressed on myeloid cells-1 (TREM-A1), which modulates TLR signaling and causes phagocytosis [96].

Phagocytosis is the process by which heterophils internalize the pathogens. Following internalization, microorganisms are entrapped in a vacuole called the phagosome, which then immediately fuses with cytoplasmic granules leading to killing of entrapped pathogens by release of anti-microbicidal peptides and proteolytic enzymes from intracellular granules [97].

Degranulation is another host defense strategy used by heterophils to kill pathogens. Upon microbial agonist stimulation, heterophils release their cytoplasmic granules at the site of infection into the external environment to kill pathogens. Another important event closely linked with extracellular degranulation is the production of heterophil extracellular traps (HET) to trap pathogens. The HET is similar to the neutrophil extracellular trap [98]. This process allows heterophils to entrap and kill bacteria extracellularly. The HET mechanism is initiated by the formation of thin extracellular fibers (5–17 nm) between pathogens and heterophils [99]. This meshwork of fibers acts as a death trap and prevent the dissemination of pathogens in the tissues. Moreover, HET also destroy pathogens by highly concentrated microbicidal substances. Finally, phagocytes also use oxidative burst to produce reactive oxygen species for microbial killing. However, unlike neutrophils, avian heterophils are mostly dependent on non-oxidative killing of pathogens as they don’t generate strong oxidative burst as compared to their mammalian analogues [100,101].

### 4.3. Dendritic Cells (DCs) of the Intestinal Immune System

DCs act as an immunological bridge between innate and adaptive immunity since these cells are the key player of antigen presentation. The intestinal DCs take up and process antigens, and present these to adaptive immune cells, which initiates a pathogen-specific immune response [102]. In mice DCs are present underneath the intestinal epithelium together with macrophages [103]. Monocytes, macrophage progenitors, and DCs express colony stimulating factor 1 receptor [104]. The intestinal DCs in human and mice can be differentiated from intestinal monocyte-derived macrophages using CD64 expression [105].

Avian DCs are less well characterized compared to human and mice DCs in which the ontogeny and heterogenous nature is extensively explored [106,107,108]. Avian DCs are defined by the expression of several markers such as CD83, CD11c, DEC205, and MHC II [109,110,111,112]. In chickens, DCs expressing CD83 and DEC205 markers are particularly abundant in the spleen, cecal tonsils, thymus, and bursa of fabricius [111]. In the chicken intestine, DCs have been described in the clusters of the GALT. Follicular DCs are located in the germinal center of the cecal tonsil, and pyloric tonsils [113,114,115]. Follicular and interdigitating DCs have also been described in the PP [116].

Immature avian DCs are equipped with a variety of TLRs to recognize invading pathogen [117]. DCs also express the CCR6 chemokine receptor. Binding of of CCR6 to this receptor induces infiltration of the DCs at the infection site to encounter antigens, due to which immature DCs transform into the mature phenotype that presents antigens to T cells. Stimulation of chicken bone marrow derived DCs with LPS results into maturation of chicken DCs, which parallels increased expression of co-stimulatory molecules CD40, CD83, CD86, and a decrease in phagocytic activity [110,118]. DCs interact with LPS of the Gram-negative bacteria through TLR4, which activates CD14 dependent endocytosis of TLR4 [119]. In addition to antigen presentation, DCs also produce cytokines such as IL1β, IL6, 1L10, IL12p35, and TNFα, and CXCL chemokines to attract other immune cells and to enhance inflammatory processes [120,121].

### 4.4. Macrophages of the Intestinal Immune System

Macrophages are a type of innate immune cells, which can be involved in the defense against bacterial infections [122]. Macrophages actively recognize, phagocytize, and kill microbes by producing microbicidal substances like nitric oxide, reactive oxygen species, proteolytic enzymes, and lysozyme [123,124,125,126,127]. Upon contact with a pathogen, macrophages become activated and transform into either M1 or M2 phenotype [128,129,130]. The polarization of the macrophages into M1 or M2 phenotypes depends upon the activation pathway and the type of cytokines they are exposed to. Exposure of macrophages to intracellular bacteria and T helper cell type 1 (Th1) cytokines (interferon and TNFα) leads to M1 polarization [131], while T helper cell type 2 (Th2) cytokines (IL4, and 13) transform macrophages into the M2 phenotype [132,133,134].

Whether this polarization also occurs in chicken macrophages is currently not clear. It has been shown, in vitro, that cultured avian macrophages can have a more M1 like phenotype as described by Peng et al. [135] for monocyte-derived macrophages, while an IL4 induced polarization towards a more M2 phenotype has also been described [136]. However, evidence for the presence of M1 and M2 macrophages in vivo is still missing.

Chicken macrophages recognize pathogens by their phagocytic receptors [137], that may be opsonic (complement receptors, Fc receptors) or non-opsonic (TLRs, mannose receptors) [138,139,140,141]. Chicken intestinal macrophages possess a range of TLRs, and hence respond accordingly to a variety of bacterial ligands such as LPS, CpG oligonucleotides, and flagellin [142,143,144,145].

Upon pathogen recognition, macrophages activate intracellular signals (mitogen activated protein kinase p38) orchestrate the innate immune response by production of cytokines (TNFα, IL1, IL10), chemokines, and nitric oxide (NO) which has antibacterial properties [146,147,148,149,150,151,152,153]. In a recent study it has been shown that HD11 cells, a macrophage-like cell line, when stimulated with inactivated poultry vaccines, showed an increase in Fc-receptor driven phagocytosis as well as NO production when activated with TLR agonists [154]. In another study, HD11 cells stimulated with inactivated *Avibacterium paragallinarium*, led to NO production and express pro-inflammatory cytokines TNFα, IL1β, and IL12p40, as well as chemokines CXCLi1 and CXCLi2 [155].

In the chicken intestine, macrophages play a vital role in antigen presentation along with DCs, as well as act as regulators and effectors of immunity. Macrophages are present in the intestinal lamina propria in chickens and reach the invasion site after IFNγ production [156]. It has been shown that IFNγ stimulation increases the antiviral and phagocytic activity of macrophages and also induces IL12 and IL18 production which drives Th1 adaptive response [157,158].

### 4.5. γδ T Cells of the Intestinal Immune System

γδ T cells are unconventional CD3^+^ T cells having a unique T cell receptor (TCR) that consists of a γ chain and a δ chain. The number of γδ T cells in blood varies between 0.5 to 10% in humans, dogs, mice and monkeys [159,160], while in chickens, cattle and pigs γδ T cells ranges from 20–50% of the total circulating T cells in blood [161,162,163,164]. Apart from blood, γδ T cells have also been reported in, for example, the intestine and spleen and their frequency depends on age, sex, and strain of chickens [165,166].

The majority of the avian γδ T cells is activated in a MHC unrestricted way unlike αβ T cells [167]. Although toll-like receptors (TLR3 and 4) are present on chicken γδ T cells along with the scavenger receptor superfamily, their role in TCR independent activation of γδ T cells in chickens is still unclear [166,168]. Recently, Karunakaran et al. showed the TCR dependent activation of human γδ T cells. After stimulating γδ T cells with phospho-antigens, molecules present inside the infected cells, γδ T cells get activated to kill the target cell [169]. Activated γδ T cells also produce IL17 and IFNγ cytokines to attract other innate immune cells [170].

The primary function of γδ T cells is to recognize stressed, transformed, and tumor cells, and their killing by perforins and granzymes [171,172]. The mode of action of γδ T cells in chickens is still unclear, but a recent study demonstrated that γδ T cells show killing of LSCC-RP9 cells [166].

γδ T cells are abundantly present in the chicken intestine. It has been shown that the percentage of γδ T cells among IEL population in the ileum of the chicken intestine remained the same during late embryonic days and early post-hatch days but started to increase from day 14 to day 21 post-hatch. As compared to other T cell subsets in the chicken intestine such as CD8αα and CD8αβ T cells, the presence of γδ T cells is lower in chickens younger than 7 days of age, but from day 14 onwards their number becomes similar to CD8αα and CD8αβ T cells [70].

## 5. Interactions between *Salmonella* and Innate Immune Cells in the Chicken Intestine

*Salmonella* serovars enter chickens via the oral route through infected feed, water, litter or through vertical transmission from infected hens, and colonize the distal part of the ileum and cecum. Intestinal epithelial and immune cells embedded in the gut epithelium provide the first protective barrier against *Salmonella*. In the lumen of the chicken gut, *Salmonella* serovars outcompete the gut microflora and make the first contact with intestinal epithelial cells [177].

As *Salmonella* are facultative intracellular bacteria, and thus can invade intestinal epithelial cells, after adherence through TLR5. The flagella and fimbriae present on the bacterial cell surface facilitate the adhesion of *Salmonella* serovars to intestinal epithelial cells [178,179]. Once attached, *Salmonella* serovars establish themselves into an intracellular niche using the protein complex T3SS-1 (type 3 secretion system-1). T3SS-1 together with its effector proteins acts as a molecular syringe, and thereby ensures the efficient invasion of *Salmonella* serovars in the host cells [180,181,182,183,184,185,186]. Several kinds of effector proteins such as SopA/B/D/E/E2, AvrA) secreted by the T3SS-1 machinery into the host cell cytoplasm, particularly SopE/E2 and SopB which activate Rho GTPase and transform inactive GDP to an active GDP bound to initiate translocation of NF-κB in the nucleus [187,188,189]. The activation of the NF-κB pathway induces the expression of pro-inflammatory cytokines such as IL18, IL1β and CXCL chemokines which attract innate immune cells such as heterophils, macrophages, and DCs to limit *Salmonella* invasion as shown in Figure 1c [94,174,175,176]. In chickens, the fate of the initial inflammatory response and consequently disease progression due to *Salmonella* serovars (systemic or gut restricted) depend on the presence or absence of a flagella, a TLR5 agonist, [190]. It is well demonstrated, both in vitro and *in vivo*, that flagellated *Salmonella* serovars like *S.* ser. Gallinarum and *S.* ser. Pullorum elicited lower or no inflammatory signal during epithelial invasion compared to flagellated *Salmonella* serovars [54,191]. Aflagellated *Salmonella* serovars cross the gut epithelial barrier using stealth strategy and, therefore, evade recognition by intestinal epithelial cells, which leads to systemic infection [191]. Moreover, mutations in the flagellin gene of flagellated *Salmonella* serovars such as *S.* ser. Typhimurium also cause rapid epithelial invasion of *Salmonella* serovars with low inflammation [54,191].

Following *Salmonella* invasion, avian heterophils reach the site of infection during the initial stages and attack the invading pathogen by producing toxic reactive oxygen species and microbicidal peptides [100,192,193,194]. An in vivo study demonstrated the influx of heterophils at the site of inflammation which resulted in resistance against *Salmonella* infection in chickens [143]. Heterophils also use extracellular traps to kill *Salmonella* serovars. Jana et al. [195] have shown that exposure of *S.* ser. Enteriditis to heterophils results in their formation within 15 min in vitro. Moreover, heterophils interact with invading *Salmonella* serovars through TLR5, which is specific for bacterial flagellin [196]. The TLR5-flagellin interaction activates the MyD88-dependent downstream signaling pathways in host cell [197,198], which activates MAPK and NF-κB and consequently leads to secretion of pro-inflammatory cytokines that causes the influx of resident and circulating immune cells at the infection site [199,200,201,202,203].

Macrophages also reach the site of infection in the initial stages of infection and recognize *Salmonella* serovars through their surface TLRs. The activated macrophages then produce NO, which is often used as a functional readout of macrophage activation, to kill *Salmonella* [204]. This inflammatory response is age dependent and is more pronounced in young chickens (up to 2 weeks of age) compared to adults [205]. It has been shown, in vitro, that HD11 macrophages induced significant NO production after 24 h of infection with chicken serum opsonized *S.* ser. Gallinarum [206].

The extent to which macrophages respond against *Salmonella* serovars is correlated with the susceptibility of chickens against the infection [207]. The phagocytic potential of macrophages against invading pathogens and their extent to present antigens differs a lot among various chicken breeds [152]. Sun et al. [208] have studied the phagocytic potential of monocyte-macrophages of the Silky and Starbro chicken lines in vitro and found that the degree of adherence of *S.* ser. Pullorum to silky monocyte-macrophages was 1.5 times higher than to those of Starbro chickens. Similarly, the phagocytic potential of the macrophages of Silky chickens was also greater than Starbro chickens following *S.* ser. Pullorum inoculation [208].

Macrophages also activate the NF-κB pathway which induces release of inflammatory factors such as the cytokines IL6, IL8, and TNFα to strengthen early inflammatory responsiveness and to promote immune defense [209,210]. Recently, it has been shown that stimulation of HD11 macrophages with *Salmonella* serovars induced the production of pro-inflammatory cytokines such as IL1β, IFNγ, and CXCLi chemokines, *in-vitro*, during initial stages of infection [206]. In the meantime, DCs present in the GALT, also reach at the invasion site, recognize the invading *Salmonella* serovar and present it to the adaptive immune cells to initiate the adaptive immune response [211].

*Salmonella* serovars can also use macrophages and dendritic cells as a vehicles for systemic dissemination of infection by using *Salmonella* pathogenicity island (SPI) T3SS-2 [212,213]. Upon successful crossing of the intestinal epithelial barrier, *Salmonella* serovars invade the underlying lymphoid tissues and subsequently get endocytosed by host macrophages [214]. Inside the macrophages, *Salmonella* serovars reside in membrane bounded vesicles known as *Salmonella* containing vesicles (SCV), where T3SS-2 with its effector proteins is rapidly expressed to promote survival and replication of *Salmonella* serovars inside the host macrophages, and prevent the lysis of invading bacteria by the host cell lysosomes [215]. It is believed that the *Salmonella* serovars are carried by the macrophages and DC via the lymphatic system to the liver and spleen, where they replicate and cause systemic infection, although there is no experimental evidence for that [216]. Macrophages not only kill the bacteria but at the same time also transmit both soluble and contact dependent signals to the resident cytotoxic intraepithelial lymphocytes (NK cells, γδ T cells, CD8^+^ αβ T cells).

NK cells embedded between the enterocytes recognize the infected intestinal epithelial cells and are activated directly upon recognition of these transformed cells. NK cells are also activated indirectly by the soluble and contact signals transmitted by macrophages [217]. Once activated NK cells release cytoplasmic granules containing perforins and granzymes to kill *Salmonella* infected cells. In addition to that, NK cells also release the cytokine IFNγ to attract other cells to initiate phagocytosis to kill *Salmonella* and *Salmonella* infected cells. NK cells are the important source of IFNγ production during initial days of *Salmonella* infection in chickens, and they play an important role in determining the fate of disease progression in chickens [218].

In newly hatched chickens, innate immunity is even more important because host defense against pathogens actually largely depends on innate immune cells [219]. NK cells play a pivotal role in *Salmonella* infection in early days of life. Meijerink et al. [220] described that immediately after entry of *Salmonella* in the chicken gut (1 day post-infection), the numbers of intraepithelial lymphocytes including NK cell subsets and CD8^+^ T cells significantly increase. It has also been demonstrated that, in chickens, degranulation of NK cells and IFNγ production, which are the functional readouts of NK cell activation, are also increased during the initial days of *S.* ser. Enteriditis infection [220]. This not only helps to kill *Salmonella*, but also attracts additional innate immune cells to avoid *Salmonella* invasion and systemic infection. *S.* ser. Enteriditis infection in 1 day old pathogen free chickens results in the production of chemokines, which leads to macrophages infiltration to the intestine within 24 h of infection [191]. Moreover, *Salmonella* infection in young chickens also gives rise to increased presence of γδ T cells, CD8^+^ cytotoxic T cells, and CD4^+^ helper T cells within one week at the site of infection, beside the killing of *Salmonella* [218,221,222,223,224]. γδ T cells recognize the *Salmonella* infected cells through cellular stress markers or phospho-antigens and cause the killing of infected host cells. Activated γδ T cells also produce immune activation mediators such as IFNγ, and IL17 cytokines to initiate Th1 response [221,222,225]. This shows that innate immune cells play an important role in young chickens in the host defense against *Salmonella* infection.

## 6. Future Prospects

Many studies underscore the importance of intestinal innate immune cells in the defense against *Salmonella* infections. Several pre- and probiotic compounds have been reported to increase the abundance of beneficial bacteria in the gut, expression of the tight junction proteins, and TLR expression by the intestinal innate immune cells [226,227,228,229,230]. Supplementation of certain carbohydrate derivatives such as glucomannan modulates the gut microbiota that inhibit the *Salmonella* colonization in the gut of broiler chickens [11]. Moreover, oligosaccharide compounds because of their structural resemblance with LPS, a TLR4 agonist, have the potential to activate innate immune cells [227]. Targeting the innate immune cells using oligosaccharide compounds, either in-ovo or post-hatch through feed formulations, could be an alternative options to strengthen immune mediated disease resistance in chickens. The recent development of novel in vitro chicken intestine models [231,232] can be used to study immune response of novel carbohydrate compounds against intestinal pathogens including *Salmonella*. This would help us to identify compounds that can enhance the immune fitness of innate immune cells, which can be used as alternatives to antibiotics. Apart from being economically beneficial for the poultry industry, it will also limit risks of AMR resistance issues in poultry, and incidence of *Salmonella* infections in humans.

## 7. Conclusions

In conclusion, the literature described shows that the innate immune system plays a pivotal role in resisting the spread of *Salmonella* in the initial stages of infection. This is especially relevant in newly hatched chickens, where the adaptive immune system is still not fully developed. Specifically targeting these innate immune cells using for example oligosaccharide compounds may be a novel strategy to strengthen the immune mediated resistance in young chickens.

## Figures and Tables

**Table 1:** Overview of functions performed by intestinal innate immune cells against invading pathogen.

Host Cell	Mediators	Functions	References
Intestinal epithelial cells	anti-microbial peptides (AMPs), Mucus, CXC chemokines, IL12, IL18, IL1β	Provide diffusive barrier to pathogen; Attract underlying innate immune cells; shape the activity of the immune cells	[173,174,175,176]
NK cells	perforins, granzymes, IFNγ, TNFα	Cytolysis of transformed and infected host cells; shape the activity of innate and adaptive immune cells	[79,84]
Heterophils	CXCLi, IL6, IL1β, cytoplasmic granules, HET	Direct Killing of invading pathogen by phagocytosis, degranulation, HET; Attract immune cells from peripheral blood and nearby organs	[56,97,98]
DCs	CXCLi1, CXCLi2, IL1β, IL10, IL6, IL12p35, TNFα	Antigen presentation to adaptive immune cells to initiate specific immune response; attract and shape the function of innate immune cells	[121]
Macrophages	CXCLi1, CXCLi2, IL10, IL12p40, IL12, IL18, TNFα, iNOS	Direct killing of pathogen by producing reactive oxygen species, nitric oxide production; Drive Th1 adaptive immune response	[146,149,152,155,157]
γδ T cells	Perforins, granzymes, IL17, IFNγ	Cytolysis of infected host cells; Attract other innate immune cells at site of infection and shape their immune function	[170,171]
